# III-Nitride Digital Alloy: Electronics and Optoelectronics Properties of the InN/GaN Ultra-Short Period Superlattice Nanostructures

**DOI:** 10.1038/s41598-017-06889-3

**Published:** 2017-07-27

**Authors:** Wei Sun, Chee-Keong Tan, Nelson Tansu

**Affiliations:** 1 Center for Photonics and Nanoelectronics, Department of Electrical and Computer Engineering, Lehigh University, Bethlehem, PA 18015 USA; 2 Department of Electrical and Computer Engineering, Clarkson University, Potsdam, NY 13699 USA

## Abstract

The III-Nitride digital alloy (DA) is comprehensively studied as a short-period superlattice nanostructure consisting of ultra-thin III-Nitride epitaxial layers. By stacking the ultra-thin III-Nitride epitaxial layers periodically, these nanostructures are expected to have comparable optoelectronic properties as the conventional III-Nitride alloys. Here we carried out numerical studies on the InGaN DA showing the tunable optoelectronic properties of the III-Nitride DA. Our study shows that the energy gap of the InGaN DA can be tuned from ~0.63 eV up to ~2.4 eV, where the thicknesses and the thickness ratio of each GaN and InN ultra-thin binary layers within the DA structure are the key factors for tuning bandgap. Correspondingly, the absorption spectra of the InGaN DA yield broad wavelength tunability which is comparable to that of bulk InGaN ternary alloy. In addition, our investigation also reveals that the electron-hole wavefunction overlaps are remarkably large in the InGaN DA structure despite the existence of strain effect and build-in polarization field. Our findings point out the potential of III-Nitride DA as an artificially engineered nanostructure for optoelectronic device applications.

## Introduction

III-Nitride materials have been extensively studied and implemented in advancing the solid-state lighting technologies^1–14^. In addition, this material platform has also attracted tremendous efforts on developing high performance active region for optoelectronic devices including detectors and solar energy convertors^15–21^. Specifically, the demand of integrating devices covering various spectral regimes on a single nitride-based material platform drives the further pursuit of III-Nitride materials with tunable band gap property^15^. The identification of InN binary alloy with narrow energy gap has enabled the access to large energy gap coverage by utilizing III-Nitride ternary alloy with various Indium composition^22^. In specific, changing the Indium composition in InGaN ternary alloy from very low In-content to InN alloy allows large bandgap tunability from ~3.4 eV (GaN) to ~0.64 eV (InN)^22,23^, which yields excellent compatibility for various optoelectronic applications.

Particularly, the InGaN ternary alloy with high In-content has been recognized for its importance in achieving optical emitting and absorbing devices in longer wavelength covering red color regime and beyond^24^. However, the experimental realization of such material system has been limited by the challenges in growing high In-content InGaN ternary alloy. Specifically, the conventional epitaxy of InGaN alloy with high In-composition results in a phase separated material system^24–30^, which leads to detrimental issues on the electronics and optoelectronics properties of this alloy. The phase separation issue in InGaN alloy system is attributed to the present epitaxial growth conditions in the state-of-the-art epitaxy tools, i.e. metalorganic chemical vapor deposition (MOCVD) and molecular beam epitaxy (MBE)^24–30^. The limitation of growing high-quality and non-phase-separated InGaN alloy with high In-content has been one of the major barriers in the realization of high performance optoelectronic devices employing Indium-rich InGaN alloys for longer wavelength applications^15–21^. On the other hand, the nanocomposite material concept has been investigated in enhancing and tuning the mechanical/electrical/optical/thermal properties for various applications^31^, yet its utilization of the multiphase solid materials still defies the purpose of having single-phase III-Nitride material system. Therefore, new strategies based on nanostructures are necessary to circumvent the barrier to achieve a non-phase separated high In-content InGaN material, and eventually achieve the ability of tuning band gap in III-Nitride platform - specifically across the entire InGaN system.

Lin and co-workers have proposed to utilize the InN/GaN short-period superlattices as low resistivity contact for GaN material^32^. Recently, the embedding of high quality monolayer InN into GaN matrix has been realized using molecular beam epitaxy technique by Yoshikawa and co-workers^33^, providing a foundation for various subsequent theoretical and experimental studies of using InN/GaN quantum wells and short-period superlattice for bandgap engineering^34–53^. Although recent studies have suggested the possibility of using III-Nitride short-period superlattice structures to vary the energy band gap^38–48^, the studies in the InN/GaN short-period superlattice are still limited up to date, in which the electronic and optoelectronic characteristics of DA structure still require significant clarifications. Previous studies have shown that the energy band of the InN/GaN quantum well and superlattices will close as the thickness of InN is thicker than 3 to 5 monolayers due to the strong built-in polarization field^41–45^. Note that the bandgap defined in those prior-mentioned works^41–45^ are the energy difference between the conduction band minimum (CBM) and the valence band maximum (VBM) in the GaN/InN/GaN band lineup. Later, Miao and co-workers predicted that the effective energy gap of a single monolayer InN/GaN quantum well is ~2.17 eV^48^. Despite the reliability of the employed First Principle method in the prior-mentioned works^38–48^, comprehensive studies on how the quantum confined effect affects the effective bandgap in the InN/GaN short-period superlattice are still needed. Moreover, previous experimental works have also shown the discrepancies between the measured optical emission and the theoretical calculated effective bandgap in the InN/GaN double heterostructures, which is still pending studies^46–52^. In addition, the experimental studies are still limited especially on the ultra-short period InN/GaN superlattices, in which both GaN and InN layers are thinner than 4 MLs^33–37,49–53^. Additional future studies on the experimental work for the InN/GaN digital alloy with both ultra-thin GaN and InN layers is necessary to confirm the findings from theoretical works.

In this work, we present a comprehensive analysis of the ultra-short period superlattice-based InN/GaN digital alloy (DA) structure as a potential alternative into the high crystalline quality In-rich InGaN material along with the tunable effective bandgap. Numerical analyses evaluate the optoelectronic properties of such InGaN DA structure. Specifically, the tunable effective bandgap property and the corresponding absorption spectra of the InGaN DA structure are calculated and presented. In addition, the electron-hole wavefunction overlap (Γ_e-h_) was analyzed for the DA structure. Our goal in this paper is to present the feasible potential of using the DA structure as an alternative to the conventional ternary alloy with comparable optoelectronic properties. Our evaluations on the optoelectronic properties of the InGaN DA pointed out the uniqueness of III-Nitride DA and its feasibility for optoelectronic applications.

## Concepts and Modeling – Digital Alloy

The concept of DA has been proposed previously to address the challenges in growing high In-content InGaAs quantum well active regions with emission wavelength in the 1200–1300 nm spectral regimes on GaAs substrates. The implementation of the short period superlattices (e.g. DA) active region has allowed the access of band gap narrowing effect for applications requiring emission wavelength beyond 1235 nm^54–59^. Specifically, both of GaAsN/InGaAs superlattices or InGaAs/InAlAs superlattices were reported to realize DA based active regions with compositions determined by the periodicity and duty cycles of the individual sub-layers. These prior works have resulted in lasing characteristics of these DA active regions in InGaAs/InAlAs DA active region for high performance telecommunication lasers^54–56^. In an analogous manner, the concept of DA can also be applied based on the III-Nitride short period superlattice structure^38–48^.

**Figure 1:**
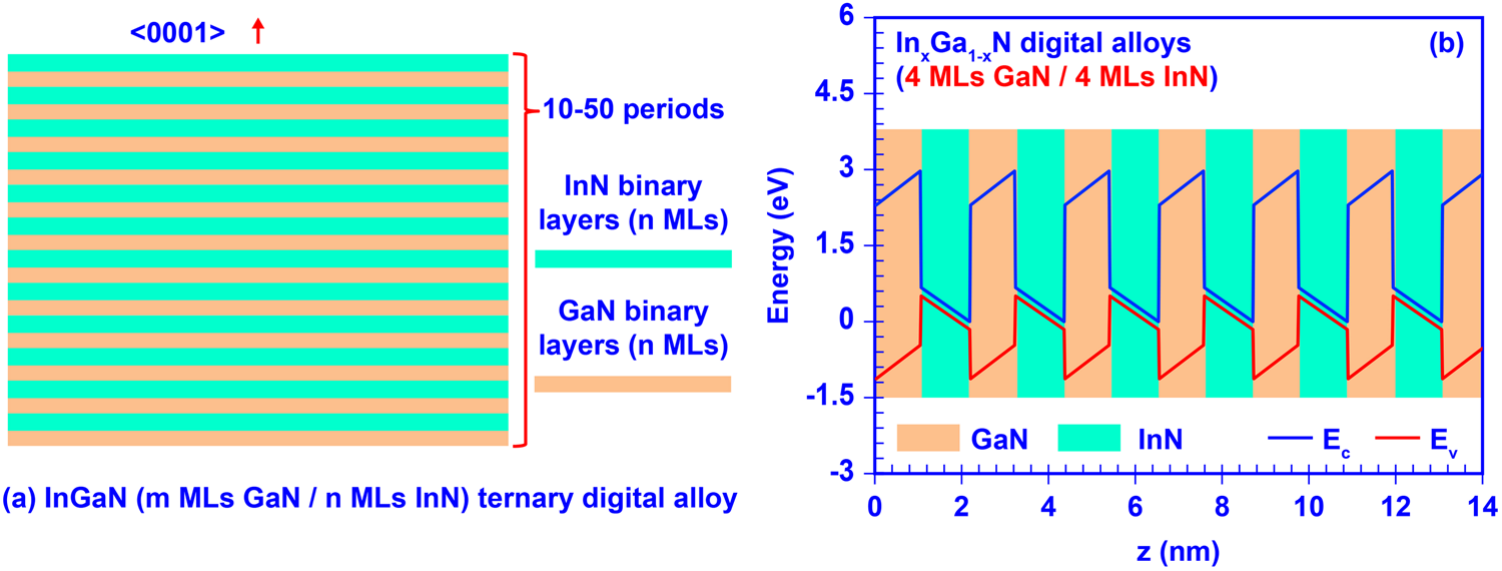
(**a**) Schematic illustration of InGaN digital alloy formed by m MLs GaN and n MLs InN ultra-thin binary layers; (**b**) Band diagram of InGaN digital alloy formed by 4 MLs GaN and 4 MLs InN binary layers.

The III-Nitride DA, specifically InGaN DA in this work, is an artificial nano-structure based on short-period GaN/InN superlattice, which is formed by alternate epitaxy of ultra-thin layers of non-phase separated GaN and InN binary alloys. Utilizing the concept of DA, the phase separation issue of the conventional ternary alloy is expected to be avoided naturally through the alternate epitaxy of high quality ultra-thin binary layers^33–37^. Figure 1(a) and (b) illustrate the schematic cross section of the InGaN DA and its band diagram respectively. The thickness of each GaN and InN ultra-thin binary layer in the InGaN DA is represented by *m* and *n* monolayers (MLs), respectively. Note that the thickness of 1 ML III-Nitride binary alloy layer along the <0001> growth direction equals to one half of its lattice constant *c*, indicating that *m* MLs GaN ultra-thin binary layer has the thickness of *m* × 2.593 Å and *n* MLs InN thin layer has its thickness of *n* × 2.851 Å^23,60^. To allow significant tunability of optoelectronic properties, m and n are limited within 1 to 4 MLs. By employing such ultra-thin GaN and InN binary layers, strong inter-well resonant coupling effect is introduced within the superlattice structure resulting in the miniband formation. Taking advantage of the resonant coupling effect, the miniband engineering can be performed by carefully designing the DA nanostructure and controlling the thickness of those ultra-thin binary layers during epitaxy process. Eventually, an effective “digital alloy” can be achieved with tunable optoelectronic properties comparable to that of bulk alloy. The experimental realization of such GaN/InN digital alloy still requires growth optimizations. However, the digital alloy structure will circumvent the fundamental limitation related to phase separation in the high In-content InGaN alloy in state-of-the-art epitaxy techniques.

**Figure 2:**
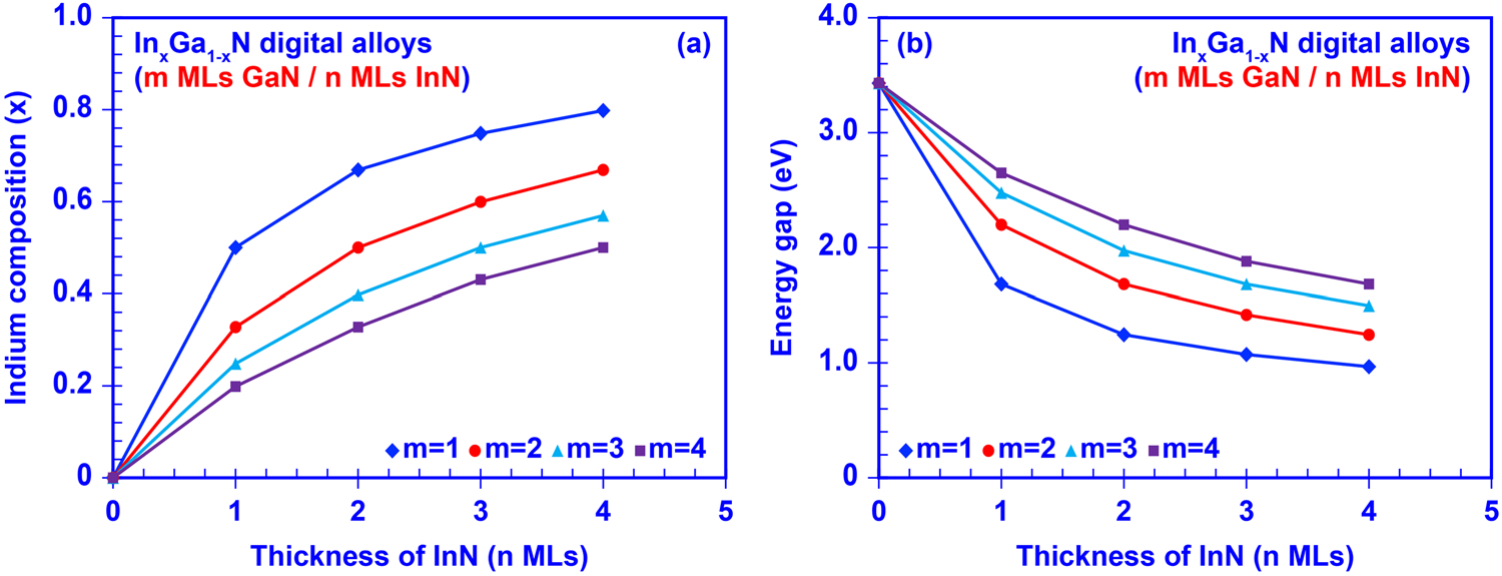
Empirical predictions of (**a**) tunability of In-Content in InGaN digital alloy and (**b**) tunability of energy gap in InGaN digital alloy.

As preliminary guidance for later numerical studies, we calculated the tunable energy gaps of InGaN DA with empirical linear interpolation including bowing parameters^60^. Figure 2(a) illustrates the tunability of Indium composition in InGaN DA. By engineering the thickness of GaN (m MLs) and InN (n MLs) ultra-thin binary layers from 1 ML to 4 MLs, the Indium composition (x) could be tuned from 20% up to 80% based on periodicity and duty cycle, e.g. x = n/(m + n). Correspondingly, the effective energy gap of InGaN DA can be tuned from 0.98 eV to 2.65 eV as shown in Fig. 2(b). For better understanding of the optoelectronic properties of the InGaN DA structure, numerical calculations were carried out on a set of 50-period InGaN DA, in which the thickness of each GaN and InN binary layers is varied from 1 ML to 4 MLs. The band lineup of the InGaN DA was modeled based on a modified Kronig-Penney model, in which the strain effect induced by lattice mismatch and built-in polarization effect attributed to both spontaneous and piezoelectric polarization are considered^60–63^. Figure 1(b) shows one example for the band diagram of the 50-period InGaN DA formed by 4 MLs GaN and 4 MLs InN ultra-thin binary layers, in which the CBM is much lower than the VBM in this DA structure attributed to the strong built-in polarization field and band edge shift induced by strain effect in the InN layer. The phenomenon shown in Fig. 1(b) indicates similar energy band lineup closing effect predicted in the previous studies^41–45^. The miniband structures, quantum confined states, effective bandgap, and carrier wavefunctions of InGaN DA were calculated by applying the transfer matrix method^64^, along with the finite differential method for the polarized energy band lineup^65^. Note that the effective energy gap defined in this paper is the energy difference between the ground confined energy states in conduction and valence sub-bands. Eventually, the absorption spectra of InGaN DA were determined based on Fermi’s golden rule^60,61^. The material parameters used in this study were obtained from previous literatures^23,60,61,65^.

## Results and Discussion

**Figure 3:**
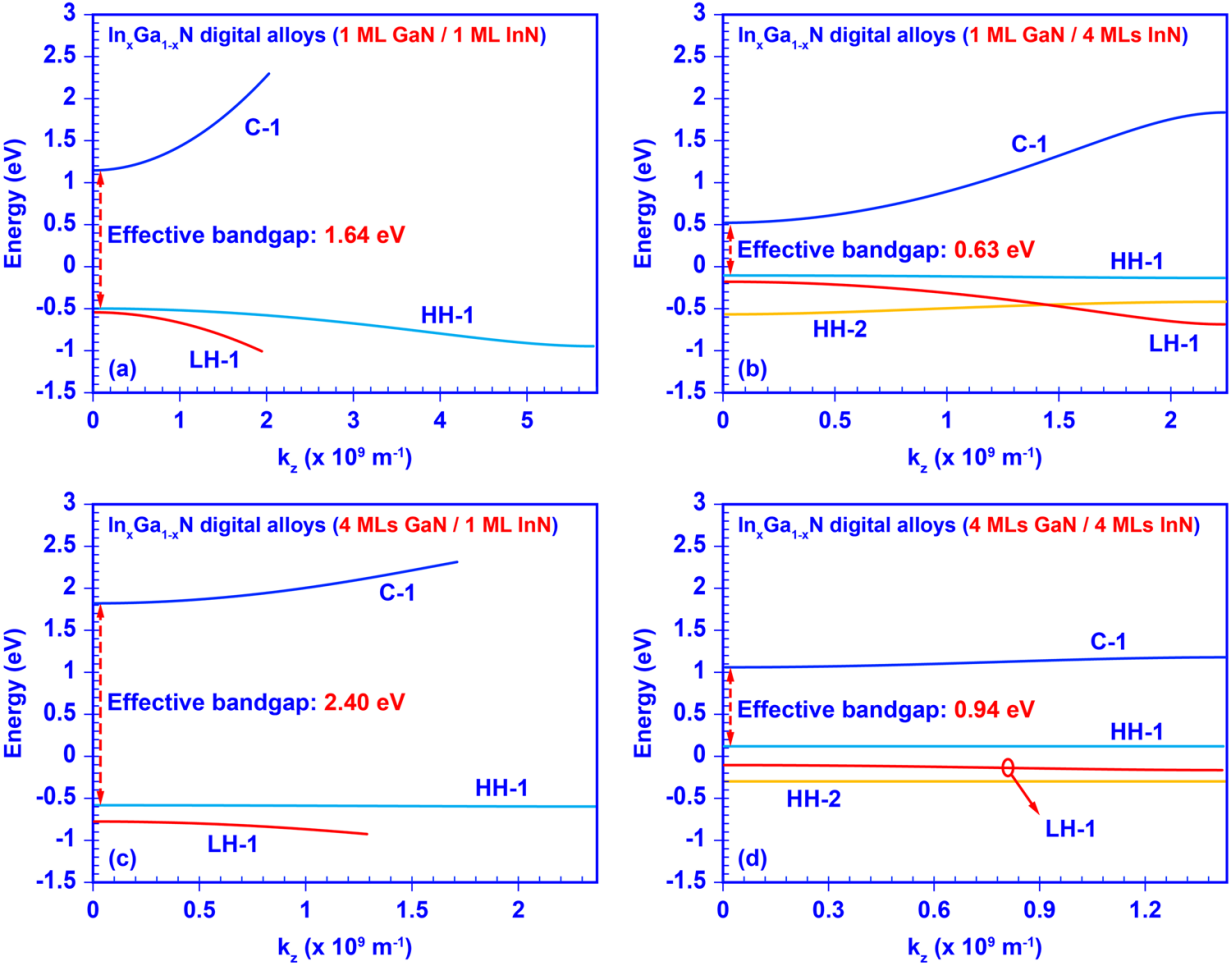
Miniband structures of the InGaN digital alloy formed by: (**a**) 1 ML GaN with 1 ML InN binary layers, (**b**) 1 ML GaN with 4 MLs InN binary layers, (**c**) 4 MLs GaN with 1 ML InN binary layers, and (**d**) 4 MLs GaN with 4 MLs InN binary layers.

Engineering the miniband structures of InGaN DA with respect to the thickness of GaN and InN ultra-thin binary layers provides an important insight in tuning the optoelectronic properties of DA structure. Figure 3 illustrates the calculated miniband structures of four typical InGaN DAs (1 MLs GaN with 1 MLs InN, 1 MLs GaN with 4 MLs InN, 4 MLs GaN with 1 MLs InN, and 4 MLs GaN with 4 MLs InN). Two notable tendencies are observed in Fig. 3 when the thickness of each GaN and InN ultra-thin layer changes. Firstly, the effective energy gap between the ground-state conduction miniband (C-1) and the ground-state valence miniband (HH-1) is reduced as the thickness of those InN binary layers increased. This phenomenon is attributed to the thicker InN binary layers that reduce the quantum confinement effect in the superlattice and therefore lower down the confined carrier states as well as the effective band gap. Secondly, the bandwidth of each miniband becomes narrower by thickening the GaN and InN binary layers. The bandwidth of the miniband is defined as the energy difference between the maximum energy and minimum energy of the miniband. As shown in Fig. 3, when the thicknesses of the GaN and InN layer increase from 1 ML to 4 MLs the inter-well resonant coupling effect within the superlattice is significantly suppressed, thus the bandwidths reduce from ~1.152 eV to ~0.116 eV for C-1 miniband, from ~0.449 eV to ~0.0001 eV for HH-1 miniband, and from ~0.451 eV to ~0.053 for the LH-1 miniband. These trends suggest that the miniband structures and optoelectronic properties of the DAs can be engineered by simply tuning the thickness of GaN and InN ultra-thin binary layers. Specifically, the effective energy gap of the InGaN digital alloys can be tuned artificially by changing the thickness of each GaN and InN thin layers. Moreover, these trends also indicate that when the thicknesses of GaN barrier layer become much larger the inter-well resonant coupling effect will become negligible leading to the degeneration of the minibands into confined-states, thus the superlattice-based DA will become the conventional multiple quantum wells (MQWs). In the case of InN/GaN DA with 1 ML thick InN, the HH-1 miniband will firstly start to degenerate when the GaN layer thickness is larger than 7 MLs. Despite the existence of the coupling of electron wavefunction for this structure with GaN thickness beyond 7 MLs, this coupling becomes very small in comparison to the case of thinner GaN barrier, which in turns result in negligible superlattice effect. This phenomenon matches well with the experimental observation by Kusakabe and co-workers^51^, in which the photoluminescence emission wavelength of 1 ML InN/n ML GaN superlattices are degenerated as the thickness of GaN layer is more than 7 MLs.

**Figure 4:**
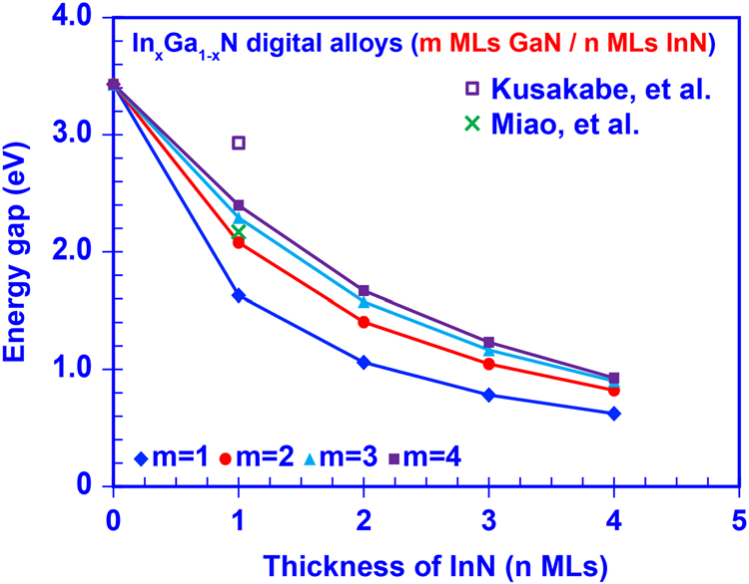
Effective energy gap of the 50-periods InGaN digital alloys formed by m MLs GaN and n MLs InN binary layers.

The tunable effective energy gaps of the InGaN DAs formed by *m* MLs GaN layers and *n* MLs InN layers are determined from the miniband calculation and illustrated in Fig. 4. Our analysis shows that the effective energy gap of InGaN DAs can be engineered from 0.63 eV (with 1 ML GaN and 4 MLs InN) to 2.4 eV (with 4 MLs GaN and 1 ML InN) because of strong inter-well resonant coupling effect and large polarization induced quantum-confined Stark effect within the GaN/InN short-period superlattices. Correspondingly, the transition wavelength was shown to be varying from ~510 nm up to ~1900 nm. Comparing Fig. 4 to our previous empirical prediction, i.e. Figure 2(b), there is a large redshift of the effective energy gap in the numerical model which is attributed to the strain and polarization effects, as well as the broadening of the miniband structure due to the thickness variation of GaN/InN ultra-thin binary layers, suggesting that our numerical model is conceptually accurate. Figure 4 shows a comparison of our calculated effective bandgap of the InGaN DA with previously reported theoretical results^48^ and experimentally measured data^51^. As show in Fig. 4, the transition energy calculated via First Principle method is ~2.17 eV for a 1 ML InN quantum well in GaN matrix^48^, which sits in the range of our calculated effective band gap for the case of InGaN DA formed by 1 ML InN with m MLs GaN. Meanwhile, the transition energy measured by photoluminescence (PL) for an InN/GaN short-period superlattice with 1 ML InN and 4 ML GaN is ~2.93 eV^51^. Note that the 0.53 eV blue shift of the effective energy gap in the PL measurement could be attributed to the imperfect InN layer embedded within the 1 ML InN/n MLs GaN superlattice structure. Suski and co-workers reported that instead of having 1 ML pure InN layer the experimental 1 ML InN/n MLs GaN superlattice samples consist of InGaN layer with average In-content of ~0.33, which could lead to the dramatic blue shift of the experimental measured effective bandgap comparing to the calculated values^47^. The broad tunability of energy gaps covered by InGaN DAs implies great potential of such III-Nitride DAs as nano-engineered structures for optoelectronics applications.

**Figure 5:**
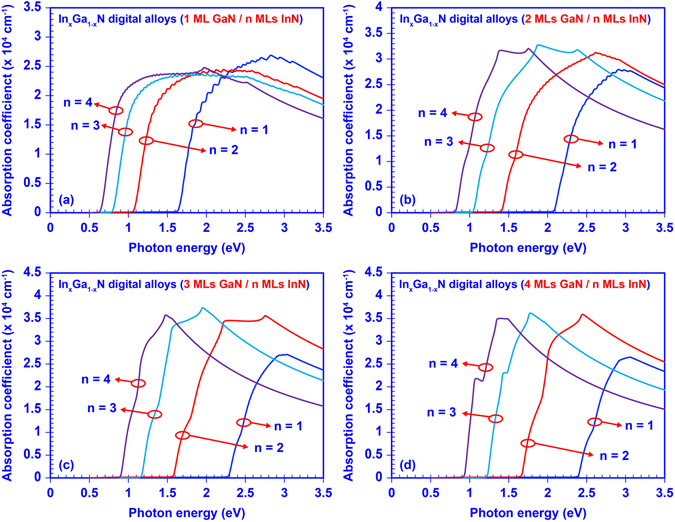
Absorption spectra of the 50-periods InGaN digital alloys: set (**a**) 1 ML GaN with 1, 2, 3, 4 MLs InN, respectively; set (**b**) 2 MLs GaN with 1, 2, 3, 4 MLs InN, respectively; set (**c**) 3 MLs GaN with 1, 2, 3, 4 MLs InN, respectively; and set (**d**) 4 MLs GaN with 1, 2, 3, 4 MLs InN, respectively.

Since InGaN DA shows substantial tunability of its effective band gap, the InGaN DA is expected to have tunable and broad absorption spectra that is also attributed to the miniband structure engineering. Figure 5 presents the calculated absorption coefficients with respect to photon energies for 16 InGaN DAs. For convenience, these 16 InGaN DAs were divided into four categories based on the thickness of GaN layer of 1 ML, 2 MLs, 3 MLs, and 4 MLs, respectively. Then in each category, the thickness of InN layer was tuned from 1 ML to 4 MLs. As shown in Fig. 5, the absorption spectra of InGaN DA are broadly saturated because of the minibands with broad bandwidth. Note that the minibands in the 50-period InGaN DA consist of 50 sub-bands that split from an original confined energy state in a non-coupled MQW system. The optical transitions are attributed to all sub-bands within the miniband of InGaN DA, which is different from that of a non-coupled MQW system. The large wavefunction overlap contributed from all sub-bands in the InGaN DA results in enhancement of the optical absorption of the active region. Therefore, a wide miniband eventually leads to a broadly saturated absorption spectrum. Besides, the cut-off wavelength of the absorption spectra shows a notable red shift when the thickness of the InN layer increases in InGaN digital alloy, which matches with the determined tunable band gap. Our analysis indicates the absorption property of InGaN DA as comparable to that of bulk InGaN ternary alloy. These findings show the importance of optimizing the structure design to obtain desirable optoelectronic properties for device applications.

**Figure 6:**
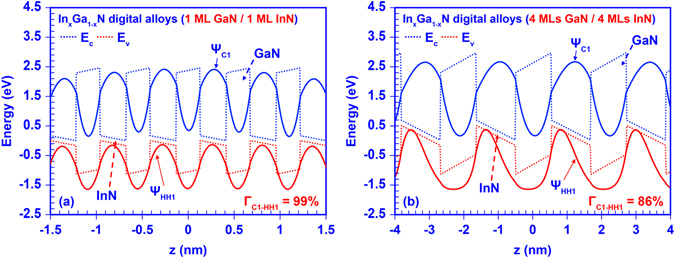
Wave functions of ground state carriers in 50-periods InGaN digital alloy formed by: (**a**) 1 ML GaN and 1 ML InN binary layers, (**b**) 4 MLs GaN and 4 MLs InN binary layers.

**Figure 7:**
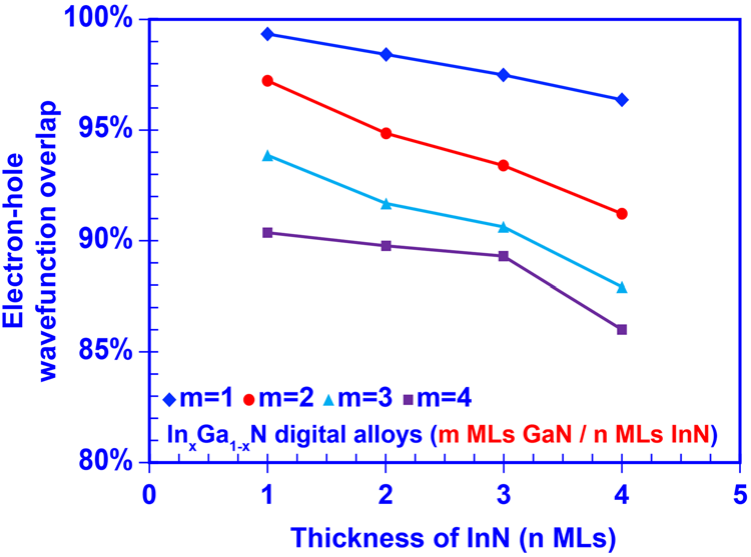
Ground state electron-hole wave function overlap as function of thickness of GaN binary layers (m MLs) and thickness of InN binary layers (n MLs).

Our numerical calculation also revealed a remarkable electron-hole wave function overlap (Γ_e-h_) property within the InGaN DA. Note that the typical electron-hole wave function overlap in a conventional polar GaN/InGaN single quantum well is limited to ~25–30% attributed to the built-in polarization field across the active region^4,9^. In contrast, large electron-hole wave function overlaps in the InGaN DA range from ~85% up to ~99%, despite the existence of the built-in polarization field. The wave functions of ground-state electron and heavy-hole are plotted in Fig. 6(a) and (b), respectively, along with the band lineups for InGaN digital alloys. As shown in Fig. 6(a), the electron and hole wavefunctions are pushed towards each other in the InGaN DA formed by 1 ML GaN and 1 ML InN ultra-thin binary layers even when the built-in polarization field still exists within the structure. Further investigation indicates that the ground state electron-hole wavefunction overlap is reduced in the InGaN DA when the thickness of each binary layer increases to 4 MLs as shown in Fig. 6(b). Our findings imply that the polarization induced charge separation issue is effectively suppressed within the InGaN DA structures by employing the strongly-coupled superlattices. Utilizing ultra-thin GaN and InN layers contributes to the large electron-hole wavefunction overlap within the DA structure. Due to the very thin GaN “barriers”, the carriers confined in InN “wells” regions couple strongly with one another through the entire superlattice. Therefore, the carrier wavefunctions no longer constrain within the wells but spread through the barriers. Thus, the envelop functions of electron and hole are determined mainly by the outer barriers region outside of the DA structure, and therefore have significantly large overlap. Meanwhile, despite of the strong built-in polarization field within InN well region, the spatial separation between electron and hole wavefunctions is extremely limited due to the ultra-thin InN layer thickness, which in turn results in the very large wavefunction overlap. Eventually, the entire InGaN DA would perform as a complete “active alloy” that exhibit comparable characteristics as conventional InGaN ternary alloy. Note that the inter-well coupling effect will be dramatically suppressed when the GaN layer become thicker leading to the carrier wavefunctions constrained within the InN layer, in turn the strong built-in polarization field will again dominantly worsen the wavefunction overlap. Figure 7 shows the calculated ground state electron-hole wavefunction overlaps of 16 different InGaN DAs. The large overlaps observed in these DAs provide a strong suggestion that this nano-structure behaves as an effective “digital alloy”.

Our numerical calculations show that the InGaN DA exhibits substantial tunability of effective band gap and optical absorption properties which are comparable to those of conventional InGaN ternary alloy. Though the valence band mixing effect in the InGaN DA is not considered in this paper, the trend and key concept discussed in this manuscript should remain. Our goal of this study is to conceptually demonstrate the potential of applying the binary alloy formed DA to overcome the barriers in the epitaxy of conventional ternary alloys and to access comparable tunability of optoelectronic properties. Further investigations considering the valence band mixing effect will be carried out via 6-band ***k*** **·** ***p*** method or other computing techniques in our future work.

## Conclusion

In summary, the III-Nitride DA is comprehensively studied based on a GaN/InN short-period superlattice for optoelectronics applications. Our findings show that the effective energy gap of a InGaN DA can be tuned from 0.63 eV to 2.4 eV by engineering the thickness of GaN and InN binary thin layers. Correspondingly, our calculation results indicate that the absorption properties of the InGaN DAs yield large wavelength coverage from ~510 nm to ~1900 nm, with their characteristics comparable to those of bulk InGaN ternary alloy. In addition, our study also suggests that the polarization induced charge separation in the conventional InGaN based active region is effectively suppressed by using very thin GaN and InN binary layers in the InGaN DA structure. Thus, the InGaN DAs exhibit remarkably large electron-hole wavefunction overlaps in the range of ~85% up to ~99%, which represent an increase of ~3–4 times over that of conventional InGaN QW. This finding suggests the strong potential for the implementation of III-Nitride DA as active region in device applications. The engineering of the GaN/InN digital alloy may provide a pathway for accessing nano-engineered materials with electronics and optoelectronics characteristics mimicking those of high In-content InGaN alloy – which would have been otherwise challenging to realize in non-phase-separated form.
